# Recent Progresses in Solution-Processed Tandem Organic and Quantum Dots Light-Emitting Diodes

**DOI:** 10.3390/molecules28010134

**Published:** 2022-12-23

**Authors:** Shu-Guang Meng, Xiao-Zhao Zhu, Dong-Ying Zhou, Liang-Sheng Liao

**Affiliations:** 1 Institute of Functional Nano & Soft Materials (FUNSOM), Soochow University, Suzhou 215123, China; 2 Jiangsu Key Laboratory for Carbon-Based Functional Materials & Devices, Soochow University, Suzhou 215123, China

**Keywords:** tandem OLED, tandem QLED, charge generation layer, solution process, orthogonal solubility, PEDOT:PSS, ZnO, stability

## Abstract

Solution processes have promising advantages of low manufacturing cost and large-scale production, potentially applied for the fabrication of organic and quantum dot light-emitting diodes (OLEDs and QLEDs). To meet the expected lifespan of OLEDs/QLEDs in practical display and lighting applications, tandem architecture by connecting multiple light-emitting units (LEUs) through a feasible intermediate connection layer (ICL) is preferred. However, the combination of tandem architecture with solution processes is still limited by the choices of obtainable ICLs due to the unsettled challenges, such as orthogonal solubility, surface wettability, interfacial corrosion, and charge injection. This review focuses on the recent progresses of solution-processed tandem OLEDs and tandem QLEDs, covers the design and fabrication of various ICLs by solution process, and provides suggestions on the future challenges of corresponding materials and devices, which are anticipated to stimulate the exploitation of the emerging light technologies.

## 1. Introduction

Due to the advantages of self-emission, high efficiency, and flexibility, organic light-emitting diodes (OLEDs) have experienced substantial development, enabling them to be a leading technology in commercial display and lighting applications [1,2,3,4,5]. Nevertheless, the industrial process for the large-scale production of OLEDs from vacuum deposition was retarded by their huge and expensive manufacturing equipment. Conversely, solution-processing methods, such as spin coating, slot die coating, blade coating, and inkjet printing, show appealing advantages in low manufacturing cost and extensive production, allowing for potential applications in industrial production [6,7,8,9,10,11]. Significant breakthroughs have been made in solution-processed materials and devices since the first polymer-based OLEDs were reported by Friend et al. [12]. Due to the efficiency limitation of polymer-based emitting materials, small-molecule organics featuring phosphorescence and thermally activated delayed fluorescence have been employed for state-of-the-art all solution-processed OLEDs [13,14,15,16,17,18,19,20], making their electroluminescence (EL) performance close to that of the evaporation method.

Despite their striking properties, solution-processed OLEDs are susceptible to external circumstances that compromise reliability. Fortunately, quantum-dot light-emitting diodes (QLEDs) have emerged as an alternative lighting technology [21,22,23,24]. Owing to quantum confinement effects, colloidal inorganic quantum dots (QDs) exhibit promising advantages, such as outstanding photostability, unique size-dependent color tunability, and high color purity [25,26,27]. The similar characteristics of solution processes to OLEDs endow QLEDs with the promising advantages of low-cost fabrication and the potential for large-area production, making them desirable candidates for display and lighting applications.

**Figure 1:**
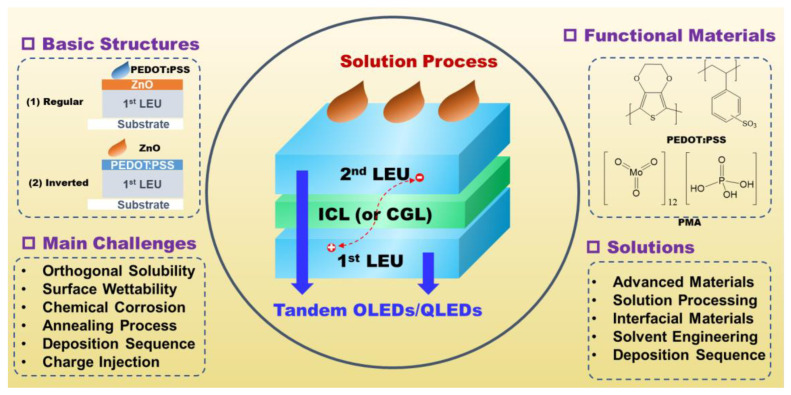
Schematic diagram of this review on the topic of solution-processed tandem organic and quantum dot light-emitting diodes (OLEDs/QLEDs); Light-emitting units (LEUs); Intermediate connection layer (ICL).

To preserve a high luminance, the multilayer emitting devices (OLEDs and QLEDs) must run at a high current density, which accelerates the degradation process of the devices. A favorable notion to lower the drive current while preserving the EL brightness is to use a tandem configuration (Figure 1), in which two (or more) light-emitting units (LEUs) are serially stacked by intermediate connection layers (ICLs, also named “charge-generation layers”, CGLs) [28,29,30,31]. Liao and Tang filed a US patent on tandem OLEDs using an ICL with an n-doped electron-transporting layer (ETL)/p-doped hole-transporting layer (HTL) structure in 2002 [28]. Bipolar charge carriers are generated by the ICL and injected into the adjacent LEUs, which gives rise to multiple photon emission from one injected hole-electron pair in tandem devices. As a result, a high brightness could be realized at a fairly low drive current, thus offering a delicate solution to extend the operational lifetime of diodes. In addition, tandem devices are potentially advantageous in constructing white light emission OLEDs [32,33]. As such, each color of emitting materials is separately confined within the individual emitting unit, thereby effectively reducing the nonradiative energy transfer among different color-mixed emitters.

However, due to the intermixing of layers, the fabrication of high-efficiency solution-processed tandem OLEDs/QLEDs remains challenging. Compared with the thermal evaporation process, the stepwise solution-based deposition of each layer has the possibility to dissolve the underneath films [34,35]. Especially in tandem OLEDs, the number of functional layers is more than that of conventional single devices, which makes the requirements for the preparation of tandem devices by solution processing much higher. Although mature and diverse ICLs have been intensively investigated in evaporated devices, there are few reports of tandem OLEDs/QLEDs using solution process techniques. Herein, we introduce the progress of solution-processed tandem OLEDs/QLEDs, with the aim of providing an overview of the design and fabrication of efficient ICLs suitable for solution processing.

## 2. ICLs for Tandem Devices

### 2.1. Type of ICLs

The EL efficiencies of tandem devices are substantially governed by the performance of the ICLs or CGLs, which are responsible for providing the generated charges into the adjacent LEUs. The design criteria for ICLs should meet (i) an efficient charge generation, (ii) high transparency, and (iii) low voltage loss [36]. The interconnecting layers of tandem devices prepared by evaporation processes can be classified into the following categories: (1) p-doped/n-doped organic semiconductors (e.g., FeCl_3_:NPB/Li:Alq, F_4_-TCNQ:m-MTDATA/Mg:Alq, etc.) [30,37]; (2) electron acceptor/n-doped ETL (e.g., HAT-CN/Li:Bphen, HAT-CN/Alq:Li, MoO_3_/TPBi:Li, MoO_3_/Mg:Bphen, WO_3_/Cs_2_CO_3_:Bphen, etc.) [38,39,40,41,42,43]; (3) organic heterojunctions (e.g., CuPc/C_60_, Pentence/C_70_, etc.) [44,45]; and (4) ultrathin conductive layer (e.g., ITO, Au, Al/Ag, etc.) [29,46]. Among them, materials such as Li and Mg used in the evaporation process are generally not applicable in the solution process, which limits the choice of materials for solution processing of ICLs. Therefore, it is urgent to find materials and processes suitable for all-solution tandem OLEDs and QLEDs.

The p-type (or electron acceptor) and n-type layers used in the evaporated ICLs usually function as a hole injection layer (HIL) and an electron injection layer (EIL) in conventional single devices, respectively. Thus, in this review, we specifically use the simplified composition of “HIL/EIL” to refer to the ICL. For solution-processed optoelectronic devices, the aqueous conductive polymer poly(3,4-ethylenedioxythiophene):poly(styrene sulfonate) (PEDOT:PSS) [47,48,49], as well as transition metal oxides (TMOs, such as MoO_3_ or WO_3_) based on sol–gel methods [50,51], are the most-used HIL materials, due to their high work function and sufficient conductivity, whereas ZnO nanoparticles with a conductive band of ~ 4.0 eV are one of the few solution processable n-type materials that can be used as EILs [52,53]. In this regard, the combination of PEDOT:PSS (or TMO) and ZnO has emerged as the most commonly used ICL. This kind of p–n junction, with a large difference in work function, proves to be an ideal ICL for the fabrication of high-performance tandem devices. Nonetheless, due to its acidic nature, PEDOT:PSS is prone to reacting with ZnO, resulting in poor morphology and degradation of the contact interface. Therefore, many efforts have been devoted to improving the properties of ICLs based on PEDOT:PSS/ZnO, which will be discussed in detail in the following section.

### 2.2. Charge Generation Mechanism

**Figure 2:**
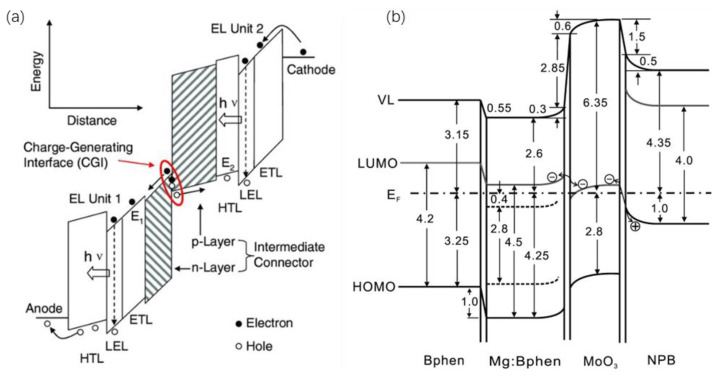
(**a**) Illustrated energy diagram of a two-EL-unit tandem OLED under forward bias. Reproduced with permission from ref. [36]. Copyright 2016, WILEY. (**b**) Illustrated energy diagram of a multilayer stack of Bphen/Mg:Bphen/MoO_3_/NPB. Reprinted with permission from ref. [42]. Copyright 2010, AIP Publishing.

The working mechanisms of various ICLs with respect to charge generation have been studied [53,54,55,56,57,58,59]. For the interconnecting layer of p-doped/n-doped organic semiconductors (Figure 2a), Liao et al. reported that charges were generated at the interface regions via temperature-independent electric field-induced electron tunneling through a thin depletion layer [36]. The p-doped and n-doped layers also facilitate efficient injection of the generated holes and electrons into the adjacent light emitting units, respectively. On the other hand, for ICLs composed of HIL/n-doped organic semiconductors, in which the HIL mainly includes TMO (such as MoO_3_ and WO_3_) and HAT-CN, charge generation is proposed to occur at the HTL/HIL interface [42], inside the HIL [32], or at both locations [60]. As shown in Figure 2b, MoO_3_/Mg:Bphen is used as an interconnecting layer [42]. Due to the high work function of MoO_3_, the HOMO electrons of NPB can be transferred to the conduction band of MoO_3_. The electrons in the conduction band of MoO_3_ can be smoothly transferred to Mg:Bphen with a reduced energy barrier caused by the energy level shift in the external electric field.

**Figure 3:**
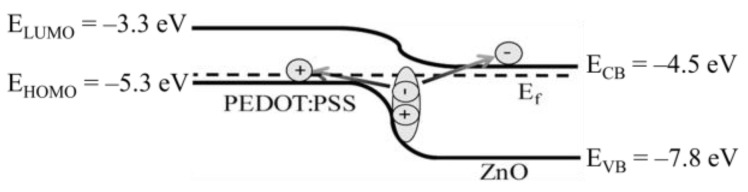
Schematic energy diagram and operational mechanism of the PEDOT:PSS/ZnO ICL. The values of the energy levels are taken from ref. [62]. Reprinted with permission from ref. [61]. Copyright 2017, American Chemical Society.

Regarding the solution-processed interconnectors, Lei et al. carefully probed the charge generation mechanism of the PEDOT:PSS/ZnO ICL by means of current density–voltage and capacitance–voltage measurements [61]. The results suggested that charges were generated at the PEDOT:PSS/ZnO heterojunction (Figure 3). The ICL-generated current can be expressed by the Richardson−Schottky thermal emission model:

(1) $$J=A^{*}T^{2}\;\exp\left[\frac{-q\left(\varphi_{B}-\sqrt{qV/4\pi \varepsilon_{i}d}\right)}{KT}\right]$$

where A* is the effective Richardson constant, *q* is the elementary charge, *φ*_B_ is the interfacial barrier, *d* is the thickness, *V* is the applied voltage, *ε*_i_ is the relative permittivity of the dielectric layer, *T* is the temperature, and *K* is the Boltzmann constant. The yielding *φ*_B_ value calculated to be 0.73 eV was consistent with the energetic difference of ca. 0.8 eV between PEDOT:PSS and ZnO. Further understanding of the operating mechanisms behind the PEDOT:PSS/ZnO ICL is anticipated with the development of novel solution-processable ICLs with improved properties.

### 2.3. Remaining Issues

**Figure 4:**
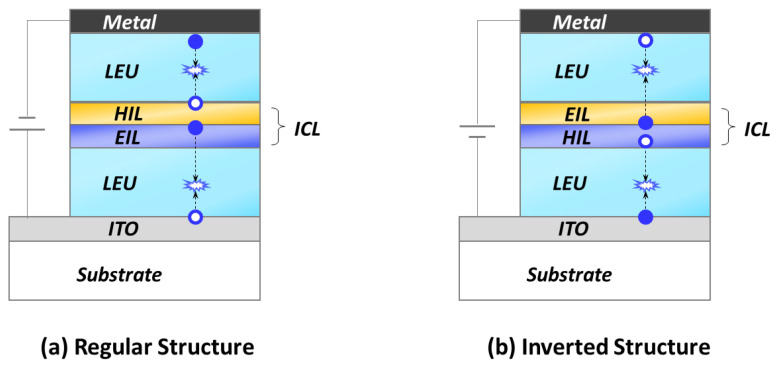
Schematic structures of tandem devices with (**a**) regular and (**b**) inverted configurations. The blue circle and dot represent the hole and electron carriers, respectively. Hole injection layer (HIL); Electron injection layer (EIL); Indium tin oxide (ITO).

The design and fabrication of efficient ICLs are highly desirable for high-performance solution-processed tandem OLEDs/QLEDs. In particular, orthogonal solubility, surface wettability, chemical corrosion, annealing temperature, and deposition sequence are crucial factors that determine the properties of a solution-processed ICL. In addition, interfacial modification needs to be taken into account to improve the charge injection process from the ICLs to the corresponding LEUs. Depending on the charge injection direction, tandem emitting devices are typically categorized into regular and inverted structures (Figure 4). In the regular structure, the bottom transparent electrode works as an anode with holes injected out, whereas, in the inverted configuration, the bottom electrode functions as a cathode with electrons injected out.

For tandem devices with regular structures, the ICL is formed by coating PEDOT:PSS onto the surface of the ZnO layer. However, the corrosive reaction of ZnO by the acidic aqueous PEDOT:PSS would cause deterioration of the ICL interface. The resultant poor wettability would not allow for the PEDOT:PSS to be uniformly spin-coated onto the ZnO. However, in the inverted structure, the ICL of PEDOT:PSS/ZnO is built by coating ZnO onto the solid PEDOT:PSS film, which mitigates the corrosion of ZnO by the aqueous PEDOT:PSS solution. Nevertheless, the large difference in the surface energies makes it challenging to uniformly deposit aqueous PEDOT:PSS onto a hydrophobic organic layer. Therefore, choosing an appropriate ICL and avoiding rinsing with the processing solvent are important in solution-processed tandem OLEDs and QLEDs.

The fabrication of well-stacked emitting multilayers also remains difficult due to the similar solubilities of emitting materials in solvents. In particular, factors such as contact angle, surface tension, vapor pressure, and solubility of the solvent need to be taken into account. Moreover, if orthogonal solvents were not selected properly for the adjacent functional layers, the underneath layers would be damaged due to solving the intermixed layers. Additionally, a low annealing temperature is required for the interconnecting layer to suppress thermal influence on the bottom LEUs.

In practice, systematic solvent engineering and finely tuned operating parameters, such as spin coating and annealing processes, are often adopted to improve film quality. For example, methanol, ethanol, propanol, and butanol have similar solubilities and can be used as dispersing solvents for ZnO NPs. The key factors affecting the quality of ZnO films include the viscosity and volatility of the solvent. During the spin coating process, the use of the relatively high viscosity of butanol will decrease the film uniformity, leading to deteriorated device performance. On the other hand, the relatively volatile methanol can lead to rapid evaporation of the solvent before the solute is uniformly dispersed and causes cracks in the film. Ethanol with moderate viscosity and volatility enables the best performance on film morphology and is therefore chosen as a suitable solvent for ZnO nanoparticles [63].

The example of choosing the solvent for ZnO clearly demonstrates the complexity and difficulty in building an efficient ICL for solution-processed tandem emitting devices. In the following sections, we will present the progress of solution-processed tandem OLEDs and QLEDs. For different configurations, regular and inverted, various ICLs are focused on. Meanwhile, the terms of orthogonal solubility, surface wettability, interfacial corrosion, and electrical transportation are discussed.

## 3. Solution-Processed Tandem OLEDs

### 3.1. ICLs for Regular Structures

As mentioned, the PEDOT:PSS/ZnO ICL is not accessible in regular device architectures (top cathode, bottom anode), where the acidic PEDOT:PSS would inevitably dissolve the underlying ZnO layer. Therefore, alternative materials to PEDOT:PSS are required to construct ICLs for regular tandem devices. In 2012, Chiba et al. reported a hybrid process of spin coating and thermal evaporation for the fabrication of ICLs with the structure of PVPy:ZnO:Cs_2_CO_3_/MoO_3_ [64], in which poly(4-vinyl pyridine) (PVPy) was utilized as a binder to improve the film morphology of the ZnO:Cs_2_CO_3_ mixture and facilitate the formation of a uniform and dense film to prevent the solvent from soaking into the first (bottom) LEU. The addition of a polymer binder can dramatically improve the film morphology without compromising the device performance. The device exhibited a sum current efficiency of 10 cd/A, with 4 cd/A contributed by the first unit and 6 cd/A by the second unit. Although the ICL exhibited good properties in stacked devices, it was limited by the vacuum deposition of MoO_3_ as the HIL.

**Figure 5:**
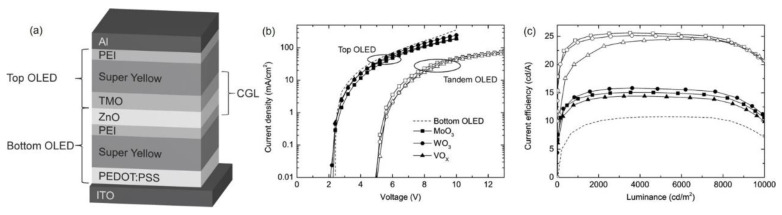
(**a**) Schematic diagram of the tandem device based on the TMO/ZnO/PEI ICL. (**b**) Current density versus voltage (J–V) characteristics and (**c**) current efficiency versus luminance (CE–L) characteristics of the corresponding single and tandem devices. Reprinted with permission from ref. [65]. Copyright 2017, American Chemical Society.

To conquer the poor solubility problems, Höfle et al. proposed the preparation of solution-processed TMOs (WO_3_, MoO_3_, or VO_x_) for interconnecting layers by the precursor method [65]. The structure of tandem devices is depicted in Figure 5a. The TMOs, including WO_3_, MoO_3_, or VO_x_, were derived from W(OEt)_6_, Mo(Oeet)_5_, and vanadium triisopropoxide precursors, respectively. Furthermore, a polyethylenimine (PEI) layer was incorporated with ZnO layers to accurately resemble the sub-OLEDs for efficient electron injection into the reference bottom LEU. The drive voltage and current efficiency of the tandem device agreed well with the sum of those for the top and bottom devices, suggesting that the TMO/ZnO/PEI-based ICL worked properly.

**Figure 6:**
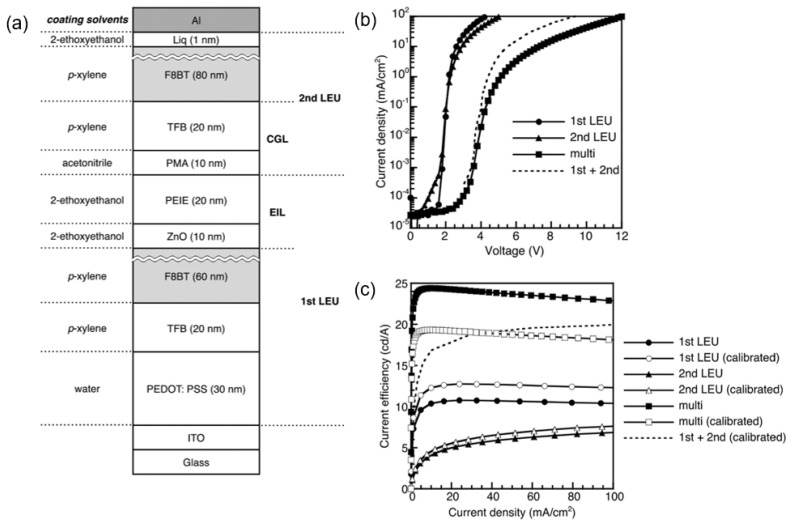
(**a**) Schematic diagram of the tandem OLED based on PMA/PEIE/ZnO ICL. (**b**) J–V characteristics and (**c**) current efficiency versus current density (CE–J) characteristics of the corresponding single and tandem OLEDs. Reproduced with permission from ref. [66]. Copyright 2014, WILEY.

The application of solution-processed TMOs solves the problem of corrosion brought by PEDOT:PSS and provides a new path for solution-processed tandem OLEDs. However, TMOs rely on the choice of precursors and subsequent annealing temperature, which increases the complexity of the process. A facile method using phosphomolybdic acid hydrate (MoO_3_)_12_·H_3_PO_4_·(H_2_O)_x_ (PMA) as an electron acceptor in the interconnecting layers was therefore reported [66]. Pu et al. used an interconnecting layer composed of ZnO/polyethylenimine ethoxylated (PEIE)/PMA/poly(9,9-dioctylfluorene-co-N-(4-butylphenyl)diphenylamine) (TFB) and realized all solution-processed tandem OLEDs (Figure 6a). To avoid the dissolution of PEIE by water or alcohols, acetonitrile was chosen as the solvent for PMA. As shown in Figure 6b, the current density–voltage (J–V) curve of the tandem device was similar to the sum of the first and second LEUs, which proved that the additional voltage drop was avoidable by the interconnecting layers. The current efficiency of tandem OLEDs (Figure 6c) calibrated by the Lambertian pattern was close to that of the sum of the first and second OLEDs.

To overcome the efficiency limitation of polymer-based emitting materials, Ohisa et al. reported all solution-processed phosphorescent tandem OLEDs with a thermally activated delayed fluorescence (TADF) host [67]. The multilayer stack of ZnO/PEIE/PMA/TFB was used as the ICL in the devices. Compared with single devices, tandem devices exhibited extremely low efficiency roll-off and enhanced operational stability. Even at a high luminance of 10,000 cd/m^2^, the external quantum efficiency (EQE) of the tandem device remained 21.9%. The findings prove that the application of PMA as an electron receptor material in ICLs is a feasible and universal strategy for solution-processed tandem OLEDs.

### 3.2. ICLs for Inverted Structures

**Figure 7:**
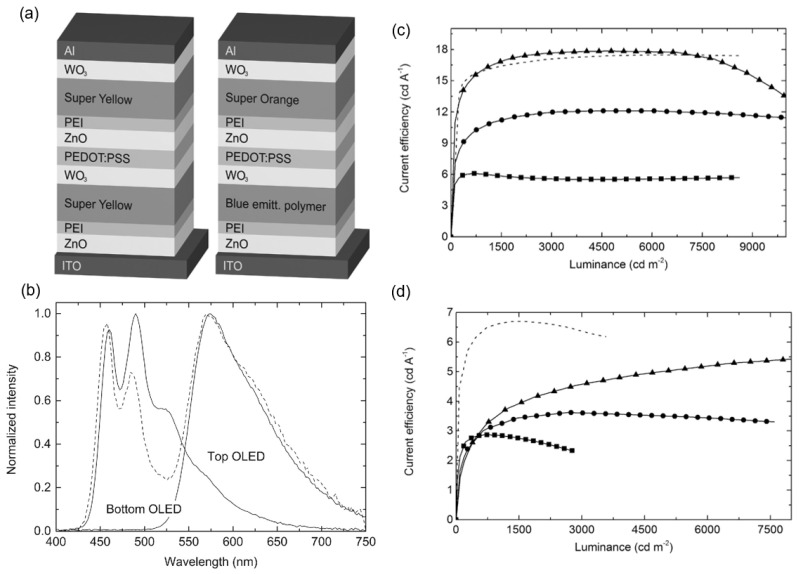
(**a**) Schematic diagram of the yellow (left) and white (right) tandem OLEDs based on the WO_3/_PEDOT:PSS/ZnO/PEI ICL. (**b**) Normalized EL spectra of the single and tandem white devices. *CE*–*L* characteristics of (**c**) yellow and (**d**) white tandem OLEDs. Reproduced with permission from ref. [68]. Copyright 2014, WILEY.

For display applications, inverted devices are desirable due to their ease of integration with n-type thin-film transistors (TFTs). Due to the mitigated reaction between ZnO and PEDOT:PSS, the composition of PEDOT:PSS/ZnO is commonly used as the ICL of inverted tandem OLEDs. In 2014, Höfle et al. first reported fully solution-processed inverted tandem OLEDs with the ICL architecture of WO_3_/PEDOT:PSS/ZnO/PEI [68]. The inverted device architecture prevents the ZnO layer from being dissolved by the acidic PEDOT:PSS solution upon deposition. It is noted that solution-deposited WO_3_ and PEI were introduced in an ICL to achieve well-matched energy alignment for better hole and electron injection, respectively. To improve the conductivity and wettability, the PEDOT:PSS layer was modified with sodium PSS and the surfactant Zonyl. Figure 7a shows the structure of a monochrome yellow and a white tandem device composing this ICL. The white emission due to the combination of orange-blue mixed EL spectra from the first and second LUEs demonstrated the feasibility of the WO_3/_PEDOT:PSS/ZnO/PEI ICL. The current efficiency (CE) of the yellow devices with two polymer light-emitting units was 18 cd/A, matching the total CE of the reference single devices, indicating that the proposed ICL structure does not cause evident efficiency loss.

**Figure 8:**
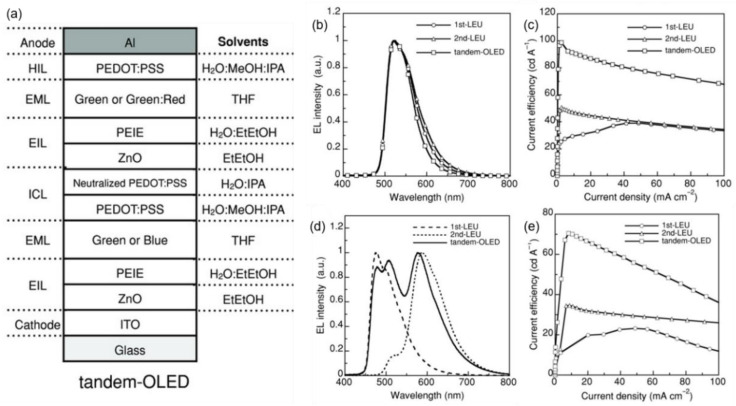
(**a**) Schematic diagram of tandem OLED based on the PEDOT:PSS/n-PEODT:PSS/ZnO/PEIE ICL. (**b**) Normalized EL spectra and (**c**) CE–J characteristics of green single and tandem devices. (**d**) Normalized EL spectra and (**e**) CE–J characteristics of bule/red single devices and white tandem device. Reproduced with permission from ref. [69]. Copyright 2015, WILEY.

Due to direct contact, the degradation of ZnO by the adjacent acid PEDOT:PSS remains inevitable. In this regard, Chiba et al. proposed a neutralized PEDOT:PSS (n-PEDOT:PSS) layer inserted between acidic PEDOT:PSS and ZnO nanoparticles (Figure 8a), thereby preventing direct contact between the two [69]. The neutralized PEDOT:PSS was achieved by the facile addition of NaOH. The film quality of the ZnO layer coated onto n-PEDOT:PSS was superior to that on pristine PEDOT:PSS. In addition, methanol and 2-propanol (IPA) were added into the aqueous PEDOT:PSS dispersion, which allowed for PEDOT:PSS to be coated uniformly over the EML. The multilayer structure PEDOT:PSS/n-PEODT:PSS/ZnO/PEIE also showed strong resistance against common organic solvents such as toluene, p-xylene, and tetrahydrofuran. A twofold increase in luminance and voltage is observed when the LEUs are stacked (Figure 8c,e). The CE of the tandem-OLED was 92.8 cd/A at 1000 cd/m^2^, equal to the sum of the corresponding efficiencies of the components first-LEU (26.7 cd/A) and second-LEU (49.4 cd/A).

Triphenylamine derivatives were generally studied as the HTL in OLEDs and perovskite solar cells [70,71,72]. Recently, Xu et al. synthesized a triphenylamine-based polymer (PTPA-P) with an ether side chain and applied it at the interface of PEDOT:PSS/ZnO [73]. The PTPA-P donor not only had a relatively shallow HOMO level (~5.0 eV) but also showed favorable insolubilities in ethanol and methanol, preventing dissolution by the subsequent processing of ZnO. Furthermore, a cross-linkable EIL of PFN-OX was introduced to avoid rinsing by the sequent solvent. The whole connecting layer of PEDOT:PSS/PTPA-P/ZnO/PFN-OX exhibited a high optical transmittance, which ensured the extraction of the light generated in the tandem PLED. Due to the higher charge generation ability of the PTPA-P/ZnO ICL, the tandem device exhibited a high current efficiency of 26.9 cd/A and an external quantum efficiency of 10.4%, which were over two times those in the corresponding single-emitting-layer device. The results verified that the PTPA-P/ZnO ICL could achieve excellent charge generation, and the generated electrons and holes could be facilely transported to the corresponding emissive layer. The solution-processed tandem OLEDs with respect to their ICL structures and current efficiencies are summarized in Table 1.

## 4. Solution-Processed Tandem QLEDs

### 4.1. ICLs for Regular Structure

Currently, QLEDs show performances competitive with that of OLEDs, while similar challenges are faced by the fabrication process of high-performance tandem QLEDs as to the OLEDs. Due to their high color purity, QLEDs feature a wide color gamut, showing a unique advantage in backlight technology for high-resolution displays. In conventional single-structure QLEDs, white light emission is usually achieved by mixing red, green, and blue QDs proportionally in a single emission layer or layers in contact [74,75]. In this way, however, white light emission can only be achieved at a specific drive voltage. The different energy levels and energy transfer between red, green, and blue QDs cause a shift in the exciton recombination region and variable light colors. The tandem structure by stacking multiple monochrome QD-based LEUs can separate red, green, and blue QDs in different LEUs and make them work independently. In this scheme of device structure, the recombination region of excitons is not affected by the driving voltage; thus, stable white light emission can be obtained.

**Figure 9:**
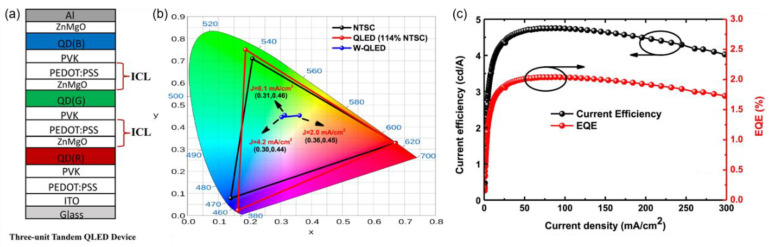
(**a**) Schematic diagram of tandem white QLEDs based on the PEDOT:PSS/ZnMgO ICL. (**b**) CIE coordinates of R/G/B monochrome QLEDs in comparison with the NTSC standard and their changes in white QLEDs under different driving current densities. (**c**) Current efficiency–luminance–external quantum efficiency (CE–J–EQE) characteristics of tandem white QLEDs. Reproduced with permission from ref. [76]. Copyright 2017, WILEY.

To achieve white tandem QLEDs with regular configurations, Zhang et al. reported an ICL composed of PEDOT:PSS/ZnMgO (Figure 9a), in which PEDOT:PSS was mixed with IPA to enhance its wettability and ZnMgO was used as an alternative to ZnO due to its improved chemical stability [76]. The CIE coordinates of blue, green and red single QLEDs were (0.16, 0.03), (0.19, 0.75) and (0.67, 0.33), respectively, showing a wide color gamut of 114% NTSC. After serial stacking, white tandem QLEDs with CIE coordinates of (0.36, 0.45) at 2 mA/cm^2^ were fabricated. Although stable white light emission was realized, the peak CE of 4.74 cd/A and the EQE of 2.0% were far lower than the theoretical sums of the three LEUs (Figure 9c). It was speculated that ICLs or other functional layers were damaged after multiple solution processing. Therefore, it is still urgent to adjust the device structure and optimize the preparation process to minimize damage and improve device performance.

**Figure 10:**
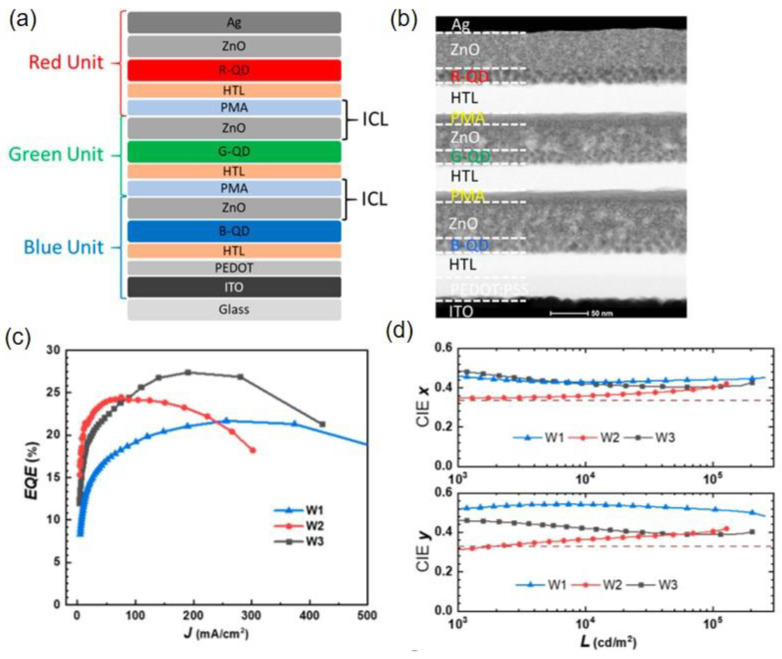
(**a**) Schematic diagram of tandem white QLEDs based on the PMA/ZnO ICL. (**b**) Cross-section transmission electron microscopy (TEM) image of tandem QLEDs. (**c**) EQE versus current efficiency (*EQE-J)* characteristics and (**d**) dependence of CIE coordinates and luminance for different tandem QLEDs. Reprinted with permission from ref. [77]. Copyright 2018, American Chemical Society.

As mentioned above, PMA has better stability than PEDOT:PSS and is more convenient to handle in the fabrication process. Jiang et al. employed PMA as the p-type electron acceptor to build a simple structure ICL of PMA/ZnO for a tandem white QLED (Figure 10a) [77]. Although the number of solution-processed functional layers exceeded 12, clear interfaces could be distinguished in the tandem white QLEDs (Figure 10b). Compared to the red and green counterparts, blue quantum dots had a deeper valence band (VB), causing a higher hole injection barrier. In this regard, different hole transporting layers, TFB, poly(vinylcarbazole) (PVK), and TFB/PVK, were used in the blue LEU to fabricate white tandem QLEDs, W1, W2, and W3, respectively. As shown in Figure 10c, the W3 device with a double-layer HTL structure TFB/PVK achieved a higher maximum EQE of 27.4% than W1 (21.1%) and W2 (24.4%). Due to the enhanced blue light emission, the W3 device exhibited stable CIE coordinates that were closer to (0.33,0.33). The results demonstrate a promising application prospect of the tandem white QLED in both display and lighting fields due to its pure emission color and high efficiency.

### 4.2. ICLs for Inverted Structure

**Figure 11:**
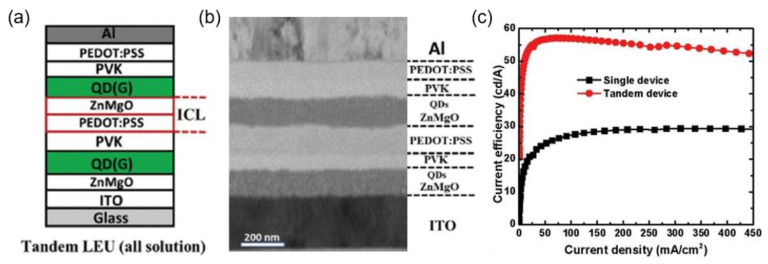
(**a**) Schematic diagram of the green tandem QLEDs based on the PEDOT:PSS/ZnMgO ICL. (**b**) Cross-section TEM image of tandem green QLEDs. (**c**) *CE–J* characteristics of single and tandem devices. Reproduced with permission from ref. [80]. Copyright 2017, WILEY.

An active matrix drive scheme is necessary in large-size and high-resolution displays [22,78,79]. To integrate QLEDs with n-type TFTs in the active matrix backplane, QLEDs with inverted device structures are preferred. Zhang et al. developed an ICL of PEDOT:PSS/ZnMgO for stacking the CdZnSeS/ZnS QD-based LUEs (Figure 11a) [80]. Compared with ZnO, Mg-doped ZnO (ZnMgO) exhibited stronger acid resistance against PEDOT:PSS. The cross-section transmission electron microscopy (TEM) image (Figure 11b) showed a distinct interface between each functional layer. Comparing the current efficiency–current density (CE–J) characteristics (Figure 11c), the highest CE of the tandem device was 57.06 cd/A, nearly twofold the 29.68 cd/A of a single device. Without an additional efficiency reduction and voltage drop, this ICL structure is considered a feasible strategy for tandem QLEDs.

**Figure 12:**
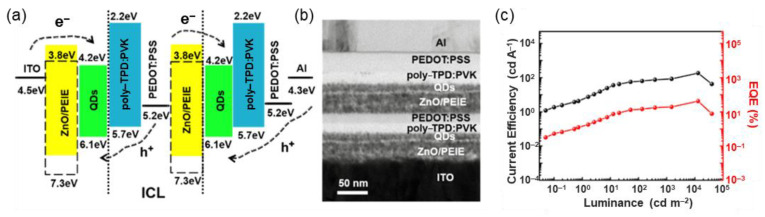
(**a**) Energy level diagram, (**b**) cross-section TEM image, and (**c**) CE–L–EQE characteristics of tandem green QLEDs based on the PEDOT:PSS/ZnO/PEIE ICL. Reprinted with permission from ref. [81]. Copyright 2019, American Chemical Society.

Due to the presence of lattice defects in ZnO nanoparticles, the direct contact between ZnO and QDs will deteriorate the EL efficiency by nonradiative recombination at the interface. Shen et al. demonstrated an inverted tandem green QLED with CdSe/ZnS quantum dots using PEDOT:PSS/ZnO/PEIE as the ICL, in which the PEIE was inserted at the interface of the interconnecting layer [81]. By introducing a PEIE-modified layer of ZnO and CdSe/ZnS QDs, a maximum CE and EQE of 183.3 cd/A and 42.2% were obtained from the proposed tandem QLED, respectively (Figure 12c). The high efficiency evidently shows the practical potential of all solution-processed QLEDs.

**Figure 13:**
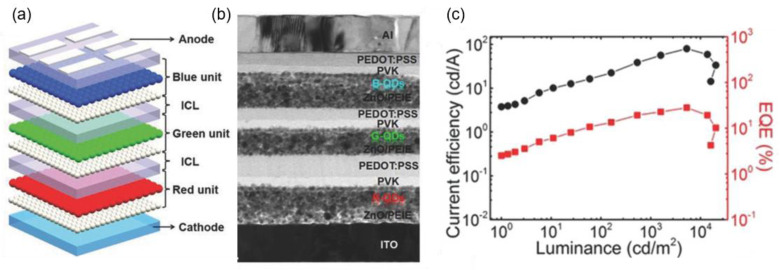
(**a**) Schematic structure diagram, (**b**) cross-sectional TEM image, (**c**) and CE–L–EQE characteristics of tandem white QLEDs based on PEDOT:PSS/ZnO/PEIE. Reproduced with permission from ref. [82]. Copyright 2018, WILEY.

Similarly, Cao et al. used the ICL of PEDOT:PSS/ZnO/PEIE to fabricate an inverted tandem white QLED [82]. The insertion of a PEIE layer between the electron transport layer ZnO and the emission layer promotes electron injection into the light-emitting layer and inhibits exciton quenching caused by defects at the ZnO interface. As a result, the tandem white QLED achieved a maximum CE and EQE of 79.9 cd/A and 28.0%, respectively (Figure 13c). This improves the record efficiency of white QLEDs, further opening the way for commercial applications of solution-processed tandem white QLEDs. The ICLs and EL parameters of solution-processed tandem QLEDs are summarized in Table 2.

## 5. Color-Tunable Solution-Processed Tandem OLED/QLED

The tandem structure, which stacks multiple LEUs, can easily double the luminous brightness, CE, and EQE. In addition, tandem structures have also been used as a strategy to realize color-tunable OLEDs/QLEDs [83,84,85]. Compared with the conventional horizontal geometric arrangement of red, green and blue subpixels, color-tunable OLEDs/QLEDs are of great significance for improving the efficiency and pixels of flat panel displays.

**Figure 14:**
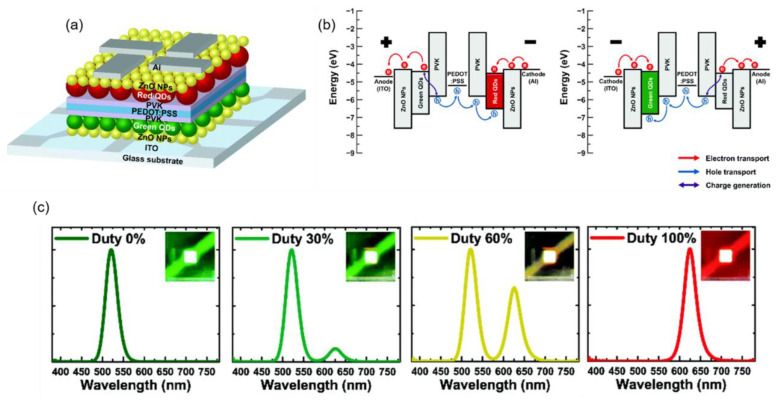
(**a**) Schematic structure diagram, (**b**) schematic of carrier transport under positive bias and negative bias, and (**c**) EL spectra of color tunable QLEDs under different duty cycles of all solution-processed color tunable QLEDs. Reproduced with permission from ref. [86]. Copyright 2020, Royal Society of Chemistry.

A schematic diagram of the structure and energy levels of a color-tunable QLED device through all solution processes is shown in Figure 14a [86]. Although the green and red QD-based LEUs were connected serially with the ICL of PEDOT:PSS/PVK, they did not work simultaneously but worked individually depending on the direction of the bias voltage. As shown in Figure 14b, both electrodes (i.e., bottom ITO and top Al) have large hole injection barriers, regardless of the positive or negative bias. When the device is forward biased, electrons can be injected from the top Al electrode (the cathode), and holes are generated at the interface of PEDOT:PSS/PVK, while the holes at the bottom ITO electrode are blocked due to the large hole injection barrier. In this case, only the red QD layer emits the light. Predictably, when a reverse voltage is applied to the device, only the green LEU works. As shown in Figure 14c, a single emission peak is observed when the duty cycle is 0% or 100%, while a mixture of two emission peaks is observed when the duty cycle is between 0% and 100%. It is predictable that color-tunable QLEDs with vertically stacked independently operated subpixels can open a promising pathway toward cost-effective ultrahigh-resolution displays.

## 6. Outlooks

A variety of ICLs for solution-processed tandem OLEDs/QLEDs have been introduced in this review. The multilayers based on PEDOT:PSS/ZnO and TMO/ZnO represent the most commonly used solution-processable ICLs in current studies. To solve the detrimental effects caused by the acidity of PEDOT:PSS, the neutralized and polymer layers have been inserted at the interface of PEDOT:PSS/ZnO; to overcome the low solubility of pure TMOs, PMA is developed as an efficient precursor; to improve the electron injection and passivate the interfacial defects, PEIE and PEI are generally incorporated with ZnO. To date, the recorded current efficiencies of the solution-processed tandem OLEDs and QLEDs are 92.8 cd/A and 183.3 cd/A, respectively. Considering the very high photoluminescence yield of the organic and QD emitting layers, there is still a large space for further improving the EL efficiency of the tandem devices from the solution process.

In terms of acceptor materials, the HILs used in ICLs are still limited to the use of PEDOT:PSS or TMO. Conversely, the HAT-CN and p-doped layers that have been intensively used in evaporated ICLs are seldom reported for solution-processed tandem devices. The realization of solution processable HAT-CN and p-doped layer would provide more choices in materials to form more efficient ICLs. In terms of device durability, few reports have focused on the operational lifetime of solution-processed tandem devices. Considering that aqueous PEDOT:PSS is generally used in solution-processed tandem OLEDs/QLEDs, the residual water due to hydrogen bonding is speculated to remain in the devices. As reported, a trace amount of water usually leads to the fast degradation of OLEDs. Therefore, the solutions that are used to solve the stability problems should be focused on. In terms of processing methods, the reported tandem OLEDs and QLEDs from solution are all fabricated by spin coating. Other solution technologies, such as slot die coating, blade coating, and inkjet printing, are more accessible to large-area production. How to combine these solution processes with the tandem structure should be investigated.

The commercial application of QLEDs has been still limited due to its short lifetime, which can be attributed to quantum dots materials, packaging technology, and devices structures [87,88]. By preparing a core–shell structure or multi-shell structure, it can effectively reduce the surface energy of quantum dots and improve their stability. An optimized packaging process can isolate QLED devices from moisture and oxygen while avoiding the harm of toxic substances such as Cd and Pb elements in quantum dots. However, the electrical degradation of QLEDs, which is caused by the corresponding increase in the current as the EL brightness increases, is difficult to solve through material design and packaging processes.

In addition to ICL, the emitting material is also an essential factor affecting the performance of light-emitting devices. As shown in this review, solution-processed tandem OLEDs are still limited by the utilization of less efficient polymer-based emitters. In the pursuit of a high efficiency and lifetime, phosphorescent and thermally activated delayed fluorescent materials are attractively desirable. On the other hand, inorganic colloidal quantum dots are intrinsically solution processable and can function as a suitable candidate of solution-processed LEDs. The dispersion solvent is the primary consideration when preparing tandem QD devices, to which the solvent of adjacent functional layers is orthogonal. In addition, the eco-friendly cadmium-free QDs would be potentially adopted in solution-processed tandem devices to overcome the toxicity problems.

Over the decades, the development of evaporated OLEDs has paved the way for solution-processed tandem devices. Although solution-processed tandem OLEDs and tandem QLEDs share similar ICL structures, colloidal QD-based LEDs generally contain less functional layers and therefore show many superior advantages compared to OLEDs. For the future, a significant job would be how to achieve the equally high efficiency and stability of tandem QLEDs with their counterpart of evaporating OLEDs. Finally, the realization of color-tunable QLEDs driven by alternating the current through tandem structures is also promising in diverse display and lighting applications.

## Figures and Tables

**Table 1:** Statistics of solution-processed tandem organic light-emitting diodes (OLEDs); Intermediate connection layer (ICL); Current efficiency (CE).

Scheme	ICL	Color	CE	Ref.
Regular	PVPy:ZnO:Cs_2_CO_3_/MoO_3_	Green	10 cd/A	[64]
	TMO(WO_3_)/PEI/ZnO	Yellow	25 cd/A	[65]
	PMA/PEIE/ZnO	Red	50 cd/A	[67]
	PMA/PEIE/ZnO	Green	19 cd/A	[66]
Inverted	WO_3_/PEDOT:PSS/ZnO/PEI	Yellow	18 cd/A	[68]
	PEDOT:PSS/n-PEODT:PSS/ZnO/PEIE	Green	94 cd/A	[69]
	PEDOT:PSS/PTPA-P/ZnO/PFN-OX	Green	27 cd/A	[73]

**Table 2:** Statistics of solution-processed tandem QLEDs.

Structure	ICL	Color	CE	Ref.
Regular	PEDOT:PSS/ZnMgO	White	5 cd/A	[76]
	PMA/ZnO	Red	28 cd/A	[77]
Inverted	PEDOT:PSS/ZnMgO	Green	57 cd/A	[80]
	PEDOT:PSS/ZnO/PEIE	Green	183 cd/A	[81]
	PEDOT:PSS/ZnO/PEIE	White	80 cd/A	[82]

## Data Availability

Not applicable.

## Sample Availability

Samples of the compounds are not available from the authors.
